# Supplementary material for: Deep learning models for forecasting dengue fever based on climate data in Vietnam
Source: PLoS Negl Trop Dis. 2022 Jun 13;16(6):e0010509. doi: 10.1371/journal.pntd.0010509 (PMC9232166; doi:10.1371/journal.pntd.0010509)
Supplement: S1 Table — Features were ranked by recursive feature elimination using a random forest regressor to rank importance. The features are listed in order from most important to least important. (DOCX) [file pntd.0010509.s001.docx]

**Table S1. Selected features for all provinces**

| Province | Features |
| --- | --- |
| Ha Noi | Max_Average_Temperature, Min_Asolute_Temperature,  Max_Daily_Rainfall, n_hours_sunshine, Average_temperature, Total_Evaporation, Min_Humidity,  Min_Average_Temperature,Total_Rainfall, Average_Humidity, Max_Asolute_Temperature, n_raining_days |
| Hai Phong | Max_Daily_Rainfall, Max_Average_Temperature, Min_Asolute_Temperature, Total_Evaporation, Total_Rainfall,  n_hours_sunshine, Max_Asolute_Temperature, Min_Average_Temperature, Min_Humidity, Average_Humidity,  Average_temperature, n_raining_days |
| Quang Nam | Average_temperature, Total_Rainfall, Min_Humidity, n_hours_sunshine,  Min_Asolute_Temperature, Average_Humidity, Min_Average_Temperature, Max_Asolute_Temperature,  Max_Average_Temperature, n_raining_days, Max_Daily_Rainfall,  Total_Evaporation |
| Quang Ngai | n_raining_days, Average_Humidity, Min_Asolute_Temperature,  Total_Evaporation, Min_Average_Temperature, Max_Daily_Rainfall,  Average_temperature, Min_Humidity, Max_Asolute_Temperature,  Total_Rainfall, n_hours_sunshine, Max_Average_Temperature |
| Phu Yen | Total_Evaporation, n_raining_days, Min_Asolute_Temperature,  Min_Humidity, Min_Average_Temperature, Max_Asolute_Temperature,  n_hours_sunshine, Max_Daily_Rainfall, Total_Rainfall, Average_temperature, Max_Average_Temperature, Average_Humidity |
| Ninh Thuan | Total_Evaporation, n_raining_days, Max_Daily_Rainfall, Max_Asolute_Temperature, Max_Average_Temperature, Min_Average_Temperature, Min_Asolute_Temperature, Total_Rainfall,  n_hours_sunshine, Average_temperature, Min_Humidity, Average_Humidity |
| Binh Thuan | Max_Average_Temperature, Total_Rainfall, Max_Daily_Rainfall, Max_Asolute_Temperature, Min_Asolute_Temperature, Min_Average_Temperature, Total_Evaporation, n_hours_sunshine,  Average_Humidity, Average_temperature, Min_Humidity, n_raining_days |
| Tay Ninh | Max_Daily_Rainfall, Average_temperature, Total_Rainfall,  Total_Evaporation, Average_Humidity, Min_Asolute_Temperature,  Min_Humidity, n_hours_sunshine, Max_Asolute_Temperature,  Max_Average_Temperature, n_raining_days, Min_Average_Temperature |
| Binh Phuoc | Total_Evaporation, Max_Asolute_Temperature, n_raining_days,  Average_Humidity, Max_Average_Temperature, Max_Daily_Rainfall,  Min_Humidity, n_hours_sunshine, Min_Average_Temperature,  Total_Rainfall, Min_Asolute_Temperature, Average_temperature |
| An Giang | Max_Daily_Rainfall, n_raining_days, Min_Asolute_Temperature,  Min_Average_Temperature, Total_Evaporation, n_hours_sunshine,  Average_temperature, Min_Humidity, Total_Rainfall, Average_Humidity, Max_Asolute_Temperature, Max_Average_Temperature |
| Tien Giang | Dengue_fever_rates, Average_Humidity, Max_Daily_Rainfall, Min_Asolute_Temperature, Total_Rainfall, Average_temperature,  n_raining_days, Total_Evaporation, Max_Asolute_Temperature,  Min_Humidity, n_hours_sunshine, Max_Average_Temperature,  Min_Average_Temperature |
| Can Tho | n_hours_sunshine, Average_temperature, Min_Asolute_Temperature,  Average_Humidity, Min_Average_Temperature, Max_Average_Temperature, n_raining_days, Max_Daily_Rainfall,  Total_Evaporation, Min_Humidity, Max_Asolute_Temperature,  Total_Rainfall |
| Tra Vinh | Max_Asolute_Temperature, Max_Daily_Rainfall, n_hours_sunshine,  Total_Rainfall, Average_Humidity, Average_temperature, Min_Asolute_Temperature, n_raining_days, Max_Average_Temperature,  Total_Evaporation, Min_Humidity, Min_Average_Temperature |
| Kien Giang | Max_Asolute_Temperature, Max_Average_Temperature, Min_Humidity,  Max_Daily_Rainfall, Min_Average_Temperature, n_raining_days,  Min_Asolute_Temperature, Total_Evaporation, n_hours_sunshine,  Average_Humidity,Average_temperature, Total_Rainfall |
| Soc Trang | n_raining_days, n_hours_sunshine, Min_Average_Temperature, Total_Evaporation, Total_Rainfall, Max_Asolute_Temperature, Min_Humidity, Max_Average_Temperature, Min_Asolute_Temperature,  Max_Daily_Rainfall, Average_temperature,  Average_Humidity |
| Bac Lieu | Max_Daily_Rainfall, Min_Asolute_Temperature, Total_Evaporation,  Max_Asolute_Temperature, Total_Rainfall, Min_Humidity,  Average_Humidity, n_hours_sunshine, Min_Average_Temperature,  n_raining_days, Average_temperature, Max_Average_Temperature |
| Ca Mau | Total_Rainfall, Max_Daily_Rainfall, Min_Humidity,  Total_Evaporation, Average_Humidity, Min_Asolute_Temperature, n_raining_days, n_hours_sunshine, Max_Average_Temperature,  Max_Asolute_Temperature, Min_Average_Temperature, Average_temperature |
| Nam Định | n_hours_sunshine, Min_Asolute_Temperature, Total_Evaporation,  Total_Rainfall, Max_Daily_Rainfall, Max_Asolute_Temperature, Max_Average_Temperature, Min_Humidity, Min_Average_Temperature,  n_raining_days, Average_Humidity, Average_temperature |
| Thái Bình | n_hours_sunshine, Max_Average_Temperature, ax_Asolute_Temperature,  Total_Evaporation, Min_Average_Temperature, Average_Humidity, Min_Asolute_Temperature, Min_Humidity, Average_temperature,  Max_Daily_Rainfall, n_raining_days, Total_Rainfall |
| Quảng Ninh | n_raining_days, n_hours_sunshine, Max_Asolute_Temperature, Total_Rainfall, Average_temperature, Total_Evaporation, Min_Humidity,  Max_Daily_Rainfall, Min_Average_Temperature, Average_Humidity, Min_Asolute_Temperature, Max_Average_Temperature |
