# Supplementary material for: Deep learning models for forecasting dengue fever based on climate data in Vietnam
Source: PLoS Negl Trop Dis. 2022 Jun 13;16(6):e0010509. doi: 10.1371/journal.pntd.0010509 (PMC9232166; doi:10.1371/journal.pntd.0010509)
Supplement: S2 Table — LSTM = long short-term memory. LSTM-ATT = attention mechanism-enhanced LSTM. (DOCX) [file pntd.0010509.s002.docx]

**Table S2. Numbers of layers and hidden sizes for LSTM, LSTM-ATT and Transformer for all provinces.**

| Province | LSTM | LSTM-ATT | Transformer |
| --- | --- | --- | --- |
| Ha Noi | 3 - 128 | 3 - 512 | 2 – 512 |
| Hai Phong | 3 - 512 | 3 - 256 | 3 – 128 |
| Quang Nam | 2 - 384 | 2 - 512 | 4 – 256 |
| Quang Ngai | 3 - 128 | 4 - 256 | 3 – 512 |
| Kon Tum | 3 - 384 | 2 - 256 | 3 – 256 |
| Phu Yen | 4 - 512 | 2 - 128 | 3 - 384 |
| Ninh Thuan | 4 - 512 | 2 - 384 | 2 – 256 |
| Binh Thuan | 3 - 256 | 3 - 256 | 4 - 128 |
| Tay Ninh | 4 - 256 | 3 - 384 | 3 – 128 |
| Binh Phuoc | 2 - 512 | 2 - 256 | 4 – 256 |
| An Giang | 4 - 384 | 3 - 384 | 4 – 128 |
| Tien Giang | 2 - 512 | 3 - 128 | 3 – 384 |
| Can Tho | 4 - 512 | 3 - 512 | 2 – 128 |
| Tra Vinh | 4 - 512 | 4 – 256 | 2 – 384 |
| Kien Giang | 3 - 512 | 3 – 256 | 2 – 256 |
| Soc Trang | 2 - 512 | 2 - 256 | 2 – 384 |
| Bac Lieu | 4 - 128 | 3 - 128 | 2 – 384 |
| Ca Mau | 3 - 384 | 3 - 256 | 2 - 384 |
| Gia Lai | 3 - 256 | 3 - 256 | 2 - 384 |
| Nam Định | 2 - 512 | 2 - 256 | 2 – 512 |
| Thái Bình | 3 - 256 | 2 - 256 | 3 – 256 |
| Quảng Ninh | 4 - 256 | 4 - 384 | 3 - 128 |

LSTM = long short-term memory. LSTM-ATT = attention mechanism-enhanced LSTM.
