# Supplementary material for: Deep learning models for forecasting dengue fever based on climate data in Vietnam
Source: PLoS Negl Trop Dis. 2022 Jun 13;16(6):e0010509. doi: 10.1371/journal.pntd.0010509 (PMC9232166; doi:10.1371/journal.pntd.0010509)
Supplement: S3 Table — p = autoregressive term. d = differencing term. q = moving average term. t = trend (n = no trend, c = constant trend, t = linear trend, ct = constant and linear trend). P = seasonal autoregressive term. D = seasonal differencing term. Q = seasonal moving average term. s = periodicity. (DOCX) [file pntd.0010509.s003.docx]

**S3 Table. Seasonal Autoregressive Integrated Moving Average model parameters.**

| Province | p | d | q | t | P | D | Q | s |
| --- | --- | --- | --- | --- | --- | --- | --- | --- |
| Ha Noi | 6 | 0 | 1 | n | 2 | 0 | 1 | 12 |
| Hai Phong | 3 | 1 | 3 | c | 1 | 0 | 2 | 12 |
| Quang Nam | 6 | 1 | 1 | n | 0 | 0 | 1 | 12 |
| Quang Ngai | 1 | 0 | 1 | ct | 1 | 0 | 2 | 6 |
| Phu Yen | 3 | 0 | 2 | t | 2 | 0 | 2 | 6 |
| Ninh Thuan | 3 | 0 | 1 | n | 2 | 0 | 1 | 6 |
| Binh Thuan | 4 | 0 | 1 | n | 2 | 1 | 0 | 12 |
| Tay Ninh | 3 | 0 | 5 | t | 3 | 0 | 2 | 12 |
| Binh Phuoc | 6 | 0 | 6 | c | 0 | 1 | 3 | 12 |
| An Giang | 4 | 0 | 3 | c | 2 | 0 | 3 | 12 |
| Tien Giang | 4 | 1 | 6 | t | 2 | 0 | 3 | 6 |
| Can Tho | 6 | 0 | 2 | t | 0 | 1 | 1 | 6 |
| Tra Vinh | 4 | 1 | 5 | n | 1 | 1 | 3 | 6 |
| Kien Giang | 1 | 0 | 3 | n | 3 | 0 | 3 | 12 |
| Soc Trang | 2 | 1 | 6 | ct | 3 | 1 | 0 | 12 |
| Bac Lieu | 2 | 1 | 6 | t | 2 | 1 | 1 | 12 |
| Ca Mau | 6 | 0 | 4 | c | 0 | 0 | 0 | 12 |
| Nam Định | 5 | 1 | 2 | n | 2 | 1 | 1 | 12 |
| Thái Bình | 6 | 1 | 1 | n | 1 | 1 | 1 | 6 |
| Quảng Ninh | 1 | 1 | 2 | c | 0 | 0 | 2 | 6 |

p = autoregressive term. d = differencing term. q = moving average term. t = trend (n = no trend, c = constant trend, t = linear trend, ct = constant and linear trend). P = seasonal autoregressive term. D = seasonal differencing term. Q = seasonal moving average term. s = periodicity.
