# Supplementary material for: Deep learning models for forecasting dengue fever based on climate data in Vietnam
Source: PLoS Negl Trop Dis. 2022 Jun 13;16(6):e0010509. doi: 10.1371/journal.pntd.0010509 (PMC9232166; doi:10.1371/journal.pntd.0010509)
Supplement: S4 Table — North, Central, and South refer to the three major geographic regions of Vietnam. (DOCX) [file pntd.0010509.s004.docx]

**Table S3. Root Mean Square Errors (RMSEs) for models assessed on 20 provinces in Vietnam.** North, Central, and South refer to the three major geographic regions of Vietnam.

| RMSE | Province/City | LSTM | LSTM-Attention | CNN | Transformer | Poisson | XGBoost | SVR | SVRLinear | SARIMA |
| --- | --- | --- | --- | --- | --- | --- | --- | --- | --- | --- |
| North | Hanoi | 7.9993 | 6.6300 | 9.1797 | 11.3006 | 17.1620 | 13.3819 | 16.6893 | 16.8776 | 18.1436 |
|  | Hai Phong | 0.4638 | 0.5286 | 0.7574 | 0.7476 | 0.9339 | 0.6570 | 6.0728 | 7.9384 | 2.5936 |
|  | Quang Ninh | 1.0101 | 0.9605 | 1.9532 | 0.9296 | 1.5772 | 1.2770 | 3.3841 | 4.0715 | 1.1748 |
|  | Nam Dinh | 0.7827 | 0.7971 | 0.9739 | 1.0083 | 0.9392 | 1.1558 | 1.4537 | 1.5777 | 0.9328 |
|  | Thai Binh | 0.6266 | 0.5967 | 0.5984 | 0.6608 | 0.6878 | 0.7384 | 0.7807 | 0.8782 | 0.6759 |
| Middle | Quang Nam | 7.3823 | 6.6964 | 6.8904 | 12.6782 | 13.5039 | 11.9901 | 13.9686 | 15.4338 | 16.4478 |
|  | Quang Ngai | 9.2875 | 8.0796 | 8.8743 | 8.8607 | 11.1132 | 9.0963 | 27.7205 | 37.6773 | 10.1812 |
|  | Phu Yen | 9.1873 | 9.5442 | 9.7655 | 12.5441 | 19.2777 | 16.2086 | 19.3285 | 20.5622 | 20.6278 |
|  | Ninh Thuan | 5.0639 | 3.9592 | 5.1395 | 8.7427 | 17.2601 | 24.8327 | 20.2742 | 12.4412 | 9.0265 |
|  | Binh Thuan | 8.3643 | 8.8255 | 8.2589 | 12.0310 | 12.9485 | 10.3023 | 13.8801 | 14.5123 | 10.1202 |
| South | Tay Ninh | 5.1226 | 3.8537 | 6.5382 | 6.5001 | 7.3497 | 9.3950 | 7.2133 | 9.4502 | 6.5995 |
|  | Binh Phuoc | 6.5774 | 7.4664 | 9.0632 | 9.6494 | 14.7958 | 12.5741 | 17.7464 | 17.5069 | 21.7310 |
|  | An Giang | 5.6985 | 3.9069 | 3.8598 | 5.4608 | 9.5022 | 8.6715 | 7.7773 | 7.9544 | 10.5036 |
|  | Tien Giang | 4.4146 | 4.0982 | 7.9115 | 5.6196 | 18.3359 | 17.6111 | 14.6476 | 16.2470 | 13.5501 |
|  | Can Tho | 3.1188 | 2.2283 | 3.9974 | 4.8661 | 8.6890 | 6.5948 | 18.5028 | 27.5181 | 9.3489 |
|  | Tra Vinh | 4.4622 | 3.8911 | 4.8201 | 4.4823 | 12.4424 | 13.6298 | 14.7521 | 14.2892 | 10.1286 |
|  | Kien Giang | 2.4604 | 2.9764 | 4.4482 | 3.8918 | 16.0698 | 16.8085 | 16.0925 | 16.4547 | 5.0786 |
|  | Soc Trang | 6.1921 | 5.8871 | 3.7250 | 4.3890 | 12.6707 | 13.9078 | 12.2272 | 11.9459 | 42.0926 |
|  | Bac Lieu | 3.4288 | 2.6520 | 2.3789 | 2.8905 | 12.3239 | 11.8407 | 10.0348 | 9.5843 | 23.8123 |
|  | Ca Mau | 4.4895 | 4.1095 | 5.4990 | 9.0425 | 14.7200 | 20.4891 | 15.2794 | 15.9739 | 17.7363 |

LSTM = long short-term memory. LSTM-ATT = attention mechanism-enhanced LSTM. CNN = convolution neural network. Poisson = Poisson regressor. XGBoost = Extreme Gradient Boosting. SVR = Support Vector Regressor with Radial Basis Kernel. SVRLinear = Support Vector Regressor with Linear Kernel. SARIMA = Seasonal Autoregressive Integrated Moving Average.
