# Supplementary material for: Deep learning models for forecasting dengue fever based on climate data in Vietnam
Source: PLoS Negl Trop Dis. 2022 Jun 13;16(6):e0010509. doi: 10.1371/journal.pntd.0010509 (PMC9232166; doi:10.1371/journal.pntd.0010509)
Supplement: S5 Table — North, Central, and South refer to the three major geographic regions of Vietnam. (DOCX) [file pntd.0010509.s005.docx]

**Table S5. Mean Absolute Errors (MAEs) for models assessed on 20 provinces in Vietnam.** North, Central, and South refer to the three major geographic regions of Vietnam.

| MAE | Province/City | LSTM | LSTM-Attention | CNN | Transformer | Poisson | XGBoost | SVR | SVRLinear | SARIMA |
| --- | --- | --- | --- | --- | --- | --- | --- | --- | --- | --- |
| North | Hanoi | 4.9258 | 3.4574 | 5.0649 | 5.6946 | 8.3966 | 7.1990 | 9.0769 | 9.5420 | 8.6372 |
|  | Hai Phong | 0.2761 | 0.3664 | 0.5380 | 0.7015 | 0.8168 | 0.4342 | 5.1962 | 7.8381 | 2.5407 |
|  | Quang Ninh | 0.6515 | 0.6142 | 1.2231 | 0.5601 | 1.3246 | 0.8762 | 2.9452 | 3.9733 | 0.7864 |
|  | Nam Dinh | 0.5558 | 0.4921 | 0.6541 | 0.7484 | 0.7957 | 0.8058 | 1.2286 | 1.4275 | 0.7284 |
|  | Thai Binh | 0.4119 | 0.4315 | 0.4277 | 0.4679 | 0.4979 | 0.4200 | 0.6638 | 0.8032 | 0.5221 |
| Central | Quang Nam | 3.7657 | 4.1162 | 4.0391 | 8.3534 | 8.7303 | 8.2158 | 9.5668 | 11.8024 | 10.5054 |
|  | Quang Ngai | 6.6988 | 6.5785 | 6.1827 | 5.9126 | 9.4420 | 6.7385 | 24.4935 | 36.9205 | 7.1117 |
|  | Phu Yen | 6.6037 | 7.3419 | 6.4329 | 10.1665 | 13.4287 | 11.9230 | 15.6076 | 17.6700 | 18.0622 |
|  | Ninh Thuan | 3.7329 | 2.8128 | 3.8754 | 5.3505 | 15.8161 | 17.6328 | 17.5656 | 9.0275 | 5.5890 |
|  | Binh Thuan | 6.6058 | 6.4948 | 6.3003 | 9.6915 | 9.9287 | 7.7552 | 11.2247 | 11.8979 | 7.2804 |
| South | Tay Ninh | 4.4049 | 2.8373 | 5.2183 | 5.3052 | 5.5165 | 6.6221 | 5.4597 | 8.2204 | 5.5852 |
|  | Binh Phuoc | 5.0196 | 5.3528 | 6.8458 | 7.5461 | 10.9571 | 10.0415 | 14.7800 | 13.7148 | 16.4401 |
|  | An Giang | 4.4617 | 3.0058 | 2.7693 | 3.7469 | 8.4761 | 7.0565 | 6.7622 | 7.0208 | 9.4234 |
|  | Tien Giang | 3.8450 | 3.3705 | 6.5885 | 4.8760 | 15.9191 | 13.5277 | 10.8930 | 14.2037 | 10.6712 |
|  | Can Tho | 2.6108 | 1.8839 | 2.9106 | 4.4692 | 6.7252 | 4.8641 | 16.7823 | 27.3701 | 8.1482 |
|  | Tra Vinh | 3.1433 | 2.7024 | 3.5278 | 4.0053 | 9.3756 | 9.4354 | 11.7660 | 11.6917 | 7.9836 |
|  | Kien Giang | 1.8483 | 2.0926 | 3.5366 | 3.1102 | 13.8586 | 12.3336 | 14.3974 | 14.6519 | 3.7648 |
|  | Soc Trang | 4.3928 | 4.5403 | 3.0837 | 3.3035 | 10.6831 | 10.3255 | 10.3103 | 10.2827 | 36.2428 |
|  | Bac Lieu | 2.8698 | 2.1599 | 2.0084 | 2.2066 | 11.4942 | 9.3986 | 9.1415 | 8.8971 | 19.5993 |
|  | Ca Mau | 3.5528 | 2.9348 | 4.5816 | 5.7097 | 12.0151 | 11.2126 | 13.1025 | 14.3807 | 16.2628 |

LSTM = long short-term memory. LSTM-ATT = attention mechanism-enhanced LSTM. CNN = convolution neural network. Poisson = Poisson regressor. XGBoost = Extreme Gradient Boosting. SVR = Support Vector Regressor with Radial Basis Kernel. SVRLinear = Support Vector Regressor with Linear Kernel. SARIMA = Seasonal Autoregressive Integrated Moving Average.
