# Supplementary figures and images for: Deep learning models for forecasting dengue fever based on climate data in Vietnam
Source: PLoS Negl Trop Dis. 2022 Jun 13;16(6):e0010509. doi: 10.1371/journal.pntd.0010509 (PMC9232166; doi:10.1371/journal.pntd.0010509)

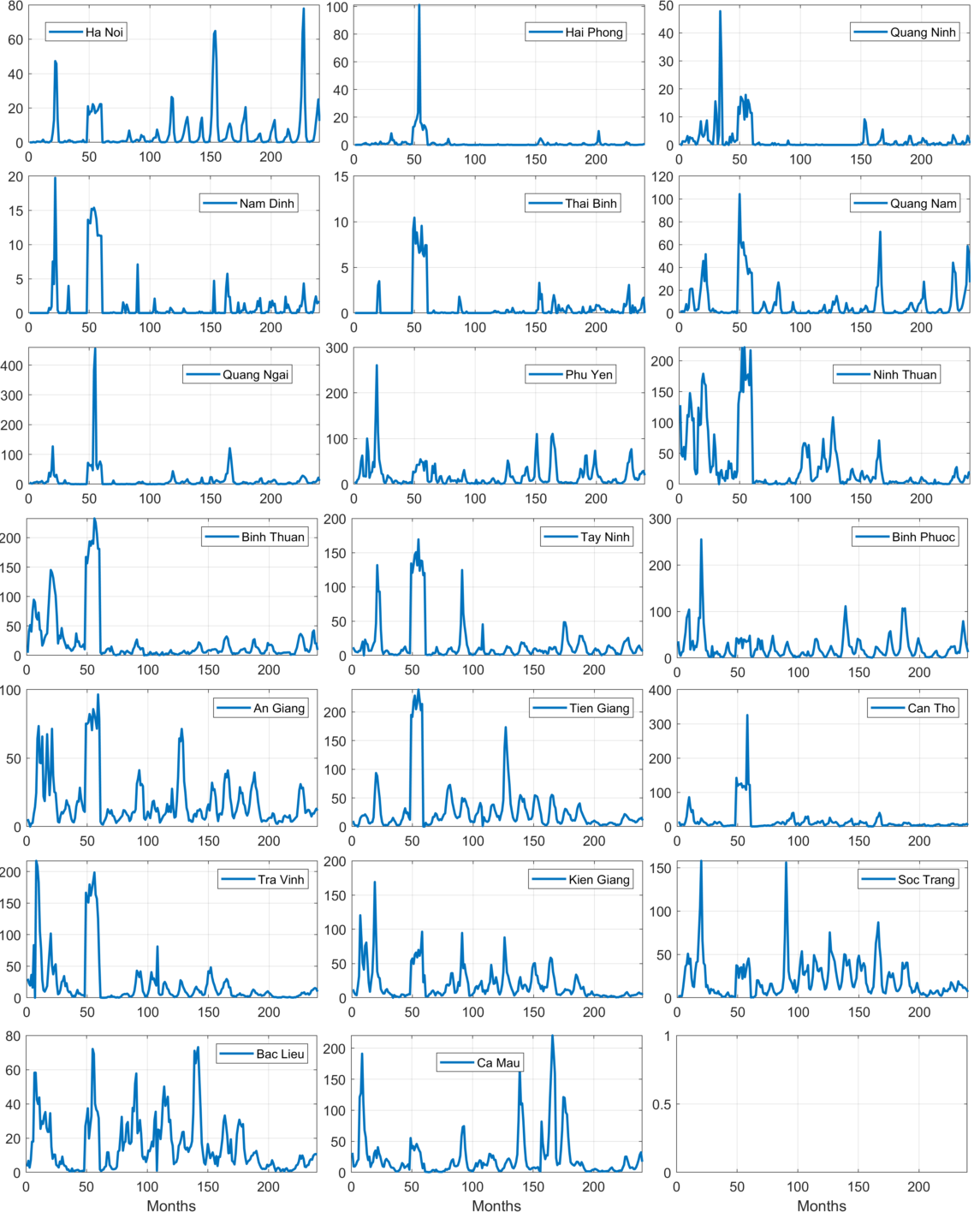

Supplement: S1 Fig — Dengue fever rates were plotted as monthly incidence per 100,000 population. (TIFF) [file pntd.0010509.s006.tiff]

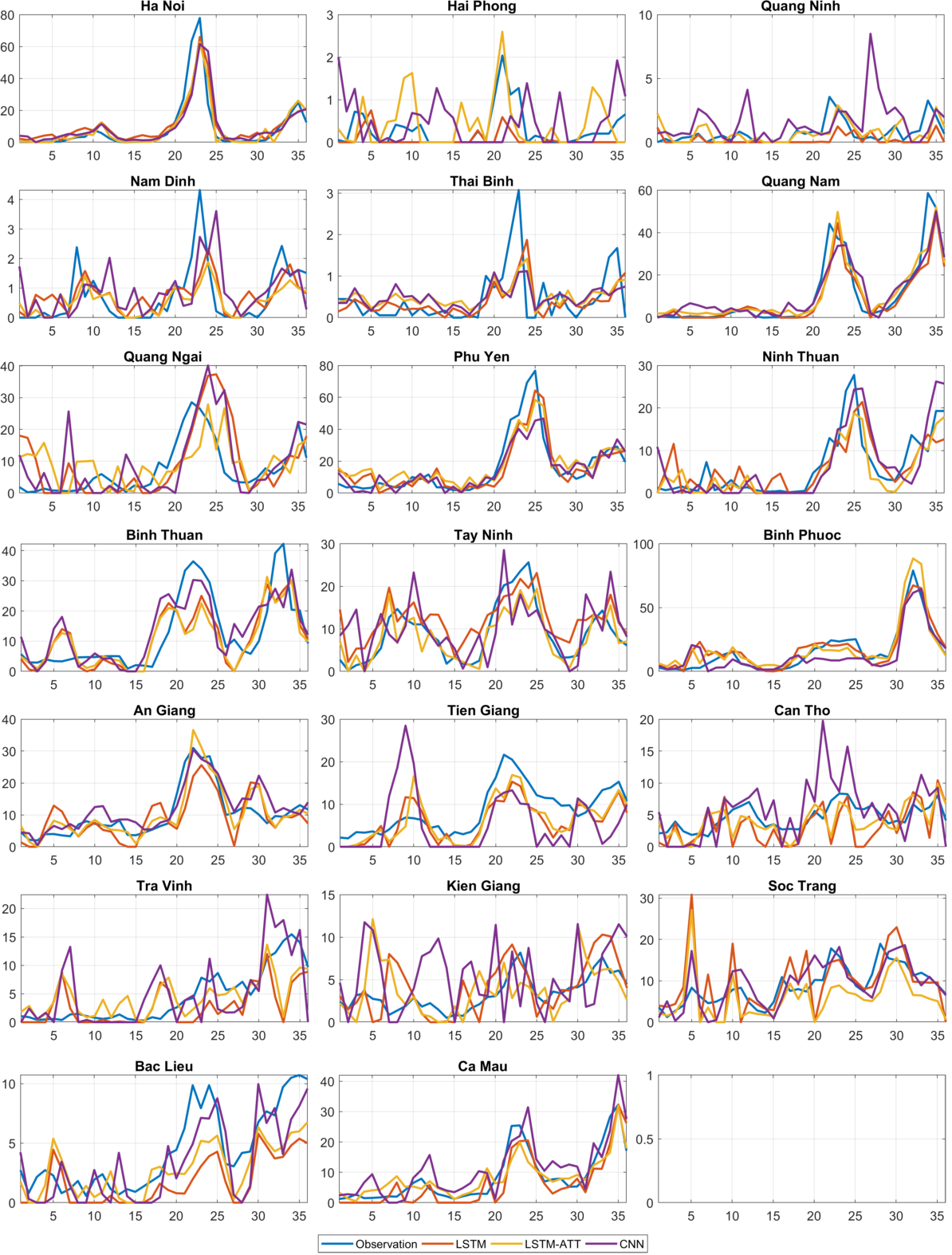

Supplement: S2 Fig — Predicted incidence rates per 100,000 population from 2014 to 2016 are shown compared to the observed incidence rates. Only the highest performing models are shown to avoid overplotting. CNN = convolutional neural network. LSTM = long short-term memory. LSTM-ATT = attention mechanism-enhanced LSTM. (TIFF) [file pntd.0010509.s007.tiff]

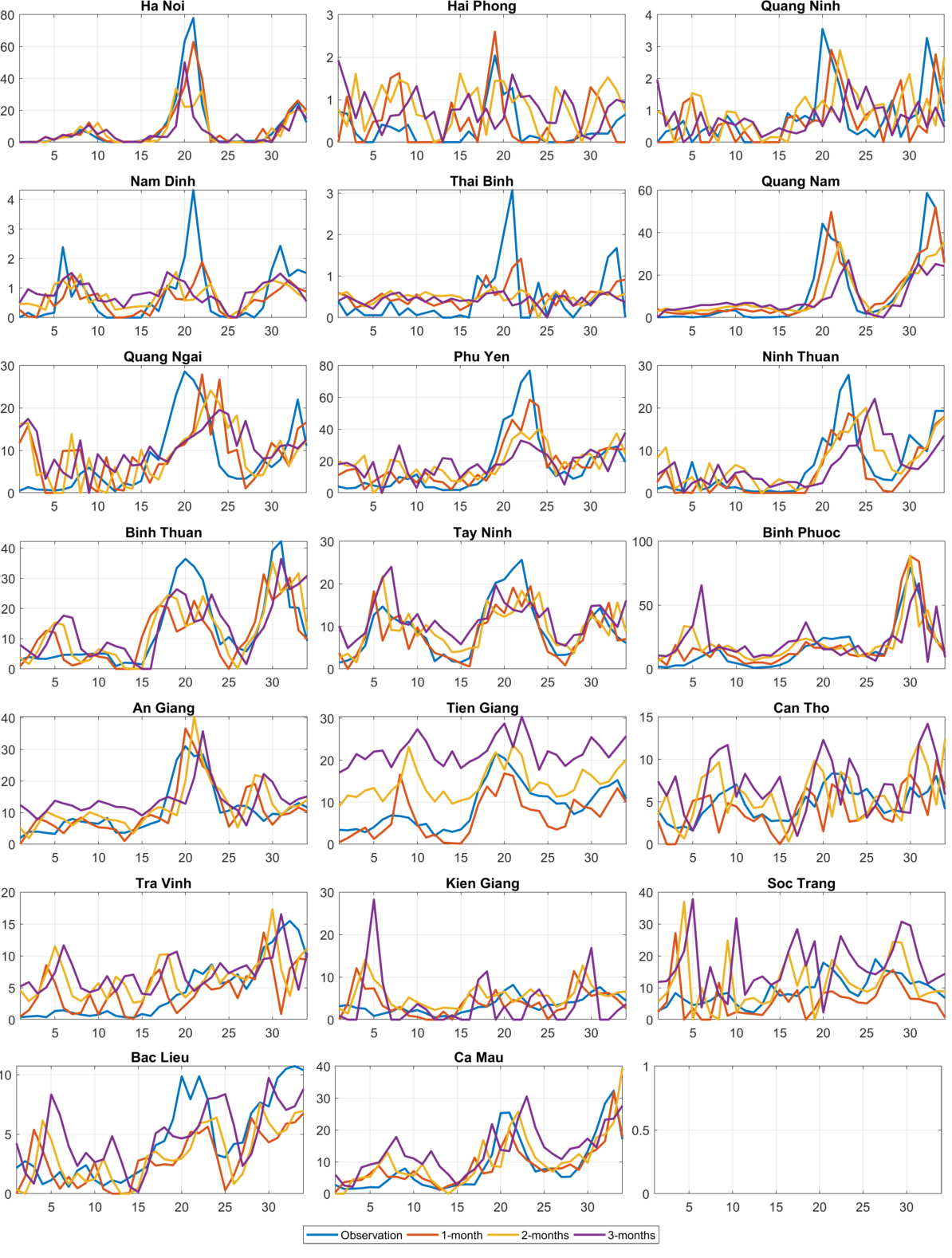

Supplement: S3 Fig — Predicted incidence rates per 100,000 population from 2014 to 2016 are shown compared to the observed incidence rates. Predicted incidence is shown for forecasts made 1–3 months ahead. LSTM-ATT = attention mechanism-enhanced LSTM. (TIFF) [file pntd.0010509.s008.tiff]

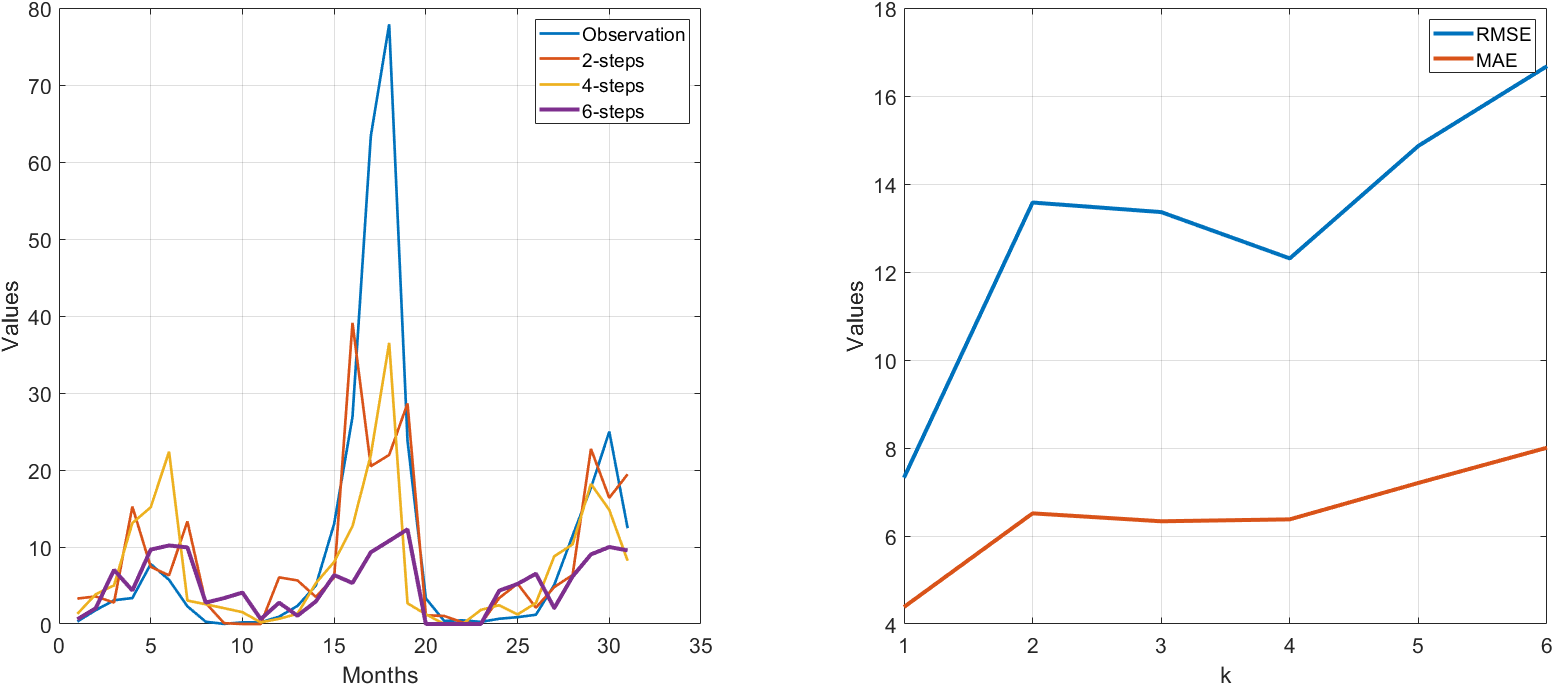

Supplement: S4 Fig — On the left, observed dengue fever incidence is plotted as well as predictions made 2, 4, and 6 steps (months) in advance. On the right, RMSE and MAE values are shown for predictions made k months in advance. (TIF) [file pntd.0010509.s009.tif]
