# Supplementary material for: Deep learning models for forecasting dengue fever based on climate data in Vietnam
Source: PLoS Negl Trop Dis. 2022 Jun 13;16(6):e0010509. doi: 10.1371/journal.pntd.0010509 (PMC9232166; doi:10.1371/journal.pntd.0010509)
Supplement: Attachment — Submitted filename: Responses to reviewers comments 26 Oct 2021.docx [file pntd.0010509.s011.docx]

**RESPONSES TO REVIEWERS’ COMMENTS**

**Deep learning models for forecasting dengue fever based on climate data in Vietnam**

Van-Hau Nguyen^1^, Tran Thi Tuyet Hanh^2^*, James Mulhall^3^, Hoang Van Minh^2^, Trung Q. Duong^3^, Van-Chien Nguyen^4^, Nguyen Thi Trang Nhung^2^, Vu Hoang Lan^2^, Hoang Ba Minh^5^, Do Cuong^6^, Nguyen Ngoc Bich^2^, Nguyen Huu Quyen^7^, Tran Nu Quy Linh^8^, Nguyen Thi Tho^9^, Ngu Duy Nghia^9^, Le Van Quoc Anh^10^, Diep Phan^11^, Quoc Viet Hung Nguyen^8^, Son T. Mai^4^

| **No.** | **Reviewers’ comments** | **Responses from authors** |
| --- | --- | --- |
| **1.** | **General comments** |  |
| **Reviewer 1** | The analysis is quite extensive and valuable, but revision is needed. Some figure can be removed, and some supplementary figure can be added for integrity of data. If the principle can be explained more clearly, like the choosing of climate factors, the article will be more persuading. Finally, the discussion which couldn’t address the objectives is unnecessary. | We carefully revise the paper following comments of reviewers. Supplementary figures are added. Deeper explanations in the climate factors are included. |
| **Reviewer 2** | No comment |  |
| **Reviewer 3** | The manuscript is original. It has great methodological strength and statistical analysis processes. The application of methodological processes in public health could have a great impact, but I consider including environmental factors of housing and the presence of breeding sites, which could strengthen the models.  The manuscript makes a good exposition of the health problem generated by dengue. Likewise, the relationship of climatic factors with dengue cases is exposed, emphasizing temperature, precipitation and others. | Yes, it would be great if we could include environmental factors of housing and the presence of breeding sites as well as the data on vector (*Aedes* mosquitos) density, which could strengthen the models. However, these data were not available at the studied provinces/cities during the studied period of past 20 years. |
| **Reviewer 4** | 47)        Dengue literature is vast. However, I was surprised not finding any reference to the important and solid prediction work performed in Thailand, for instance. Check out:  - Reich et al. (2016) Challenges in real-time prediction of infectious disease- a case study of dengue in Thailand - Lauer et al. (2018) Prospective forecasts of annual DHF incidence in Thailand, 2010-2014 - Kiang et al. (2021) Incorporating human mobility data improves forecasts of dengue fever in Thailand And the multi-model challenge of forecasting dengue in Puerto Rico: - Johansson et al. (2019) An open challenge to advance probabilistic forecasting for dengue epidemics. | References to some of the noted DF prediction studies have now been added. Specifically, the Lauer et al. (2018) study is now mentioned in the introduction, the Lauer et al (2018) and Johansson et al. (2019) studies are mentioned in the discussion, and the Reich et. al (2016) study is now mentioned in the limitations. |
|  | 48)     Writing should be improved for clarity and correctness. Several points are mentioned below. 49)     Content is mixed between sections, making reading more difficult. Several instances are mentioned below. 50)     Some analyses should be enriched or extended. See points 6 and 18. 51)     The subject and approach are interesting and relevant. Improving analyses as pointed above, improving presentation, and enriching the discussion would translate in a solid, clear, and relevant paper. | We rewrite many parts of the paper for improving clarity. All the comments from reviewers are carefully considered and changes are made throughout the paper. |
| **Reviewer 5** | No comment |  |
| **Reviewer 6** | The authors have compared current deep learning models to forecast Dengue Fever in three regions of Vietnam- CNNs, Transformer, and long short-term memory (LSTM); and they have successfully developed an attention mechanism- enhanced LSTM model (LSTM-ATT) that outperformed the previous modelling methods. I have mixed feeling about this paper. On one hand, the work is relatively impressive, with extensive datasets spanning 19 years, from 1997 to 2016, and top-notch competing deep learning methods were simultaneously tested. This study is bringing novel information on DF forecasting deep learning methods, and the results found can significantly improve current capacity to appropriately respond to predicted DF outbreaks within 1 and 3 months of actual outbreaks. However, there are some aspects that the authors may need to address to improve the overall quality of the manuscript.  My first concern is about the apparent lack of information in the materials and methods section. The study area includes 20 provinces of three main regions in Vietnam, however geographical description of the three main regions studied are not provided; additional detailed description of the 12 meteorological factors used as predictors is needed, a first description of the prediction accuracy metrics “specificity, sensitivity, true positive, etc.” is needed to improve further data comparison and interpretation. | Descriptions of the different geographical regions of Vietnam have been added, as well as more detailed descriptions of the 12 meteorological factors. Specificity, sensitivity, accuracy, and precision are now defined with equations in the methods section. |
|  |  |  |
|  | Reading the result section, I noticed some relevant information seems to be lacking, which may improve the interpretation and significance of the work. Fig 1 depicts predicted incidences vs. real incidence as part of the 1-step forecasting accuracy, there is need of statistical values, e.g. 95% credible intervals, or IC values for the real incidence line depicted on the graphs. The Table S1 provide a list of the top 2 geometrical factors that ranked best among 12 factors evaluated per each province. Actual rank values could be added for each selected factor for data completeness. | In our work, we use Recursive Feature Elimination (REF) with Random Forest as an evaluator to choose the best climate factors for Deep Learning models. Table S1 provides the full feature ranking for each province. We choose the top 2 features as input for DL models.  Unlike other traditional statistical methods, Deep Learning methods like LSTM do not provide confidence intervals for prediction results. |
|  | Additionally, not all relevant competing papers were discussed- especially one that tested a super ensemble forecasted method in the entire country of Vietnam, this paper was published very recently (Colon-Gonzales F., et al. March 2021) and its findings could inform the current study. So, although the present study could be the first-to adapt deep learning methods to forecast DF based on climate factors, other work has been done with similar outcomes and in a more complete dataset including the 63 provinces of Vietnam. | Thank you for bringing this paper to our attention, we had not come across it before. A discussion of the Colon-Gonzales et al. (2021) paper has been added. |
|  | Information on the authors' Institutional Review Board or an equivalent committee approval for research involving human participants may be needed. A brief overview of the ethics reporting should be provided in the Methods. | This study was approved and managed by the Hanoi University of Public Health. The study only involved analysing secondary data on dengue fever cases and climate factors including temperature, precipitation, humidity, evaporation, and sunshine hours. No human participants were actually involved in this study. Thus, ethical approval was not required. This is now mentioned in the Methods. |
|  | Under 2.1 Vietnam. A brief description of main geographic/climate characteristics of the three regions studied in Vietnam is needed, this will provide readers with a better idea of the importance to include provinces representing these regions in Vietnam. It will also facilitate the interpretation of the results when different predictions are obtained in each region and climate factors are driving the differences. | Vietnam has three main history, geographic and cultural regions, including Northern, Central and Southern Vietnam. Each region consists of subregions, with considerable cultural and climate differences originating from each subregions.  Northern Vietnam has a [humid subtropical climate](https://en.wikipedia.org/wiki/Humid_subtropical_climate), with a full four seasons, with much cooler temperatures than in the South (which has a [tropical savanna](https://en.wikipedia.org/wiki/Tropical_savanna) climate). Winters in the North that can get quite cold, sometimes with frost and even snowfall. Snow can even be found to an extent up in the mountains of the extreme Northern regions such as in Sapa and Lang Son province in recent years. Southern Vietnam, is usually much hotter and has only two main seasons: a dry season and a rainy season. |
| **Reviewer 7** | The study is original and revealed the usefulness of deep learning models for climate-based Dengue Fever forecasting, in predicting epidemics before three months with the employment of environmental data. It is very well written and the authors included limitations of the methods in the discussion section. |  |
| **2.** | **Introduction** |  |
| **Reviewer 1** | No comment |  |
| **Reviewer 2** | No comment |  |
| **Reviewer 3** | No comment |  |
| **Reviewer 4** | 24) Line 74: it is rather unusual to write the abbreviation of the genus between brackets. Instead, the usual is to abbreviate after the first time it is mentioned. | Thank you, this was amended. |
|  | 29)     Lines 130-1: have LSTM been used elsewhere for dengue or other vector-borne diseases? Mentioning it would provide a richer context. | LSTM has been used for DF prediction in some countries like Singapore and China. These works have been included and discussed in the paper. However, to the best of our knowledge, it has not been applied for DF studies in Vietnam before. |
|  | 1) The objective is clearly stated. However, it could be put into more relevance if the last two paragraphs of the Introduction were reorganized. | Thank you for your advice. The sentence “This study aimed to develop an accurate prediction model for DF in Vietnam, using a wide range of climate factors as input variables” is now re-allocated as the last sentence of the 2^nd^ last paragraph. |
|  | 25)     Lines 81-4 and 153-5: reference is missing supporting claim. Additionally, the effects of climate change on dengue transmission is quite disputed and seems to strongly depend on the region and the methodology and criteria of analysis. | Appropriate reference was added to support the claim as you advised:  4. Tuyet-Hanh TT, Ha VN, Hoang VM, Le TTH, Nguyen TTN, Tran MK, et al. Health Vulnerability and Adaptation Assessment in Viet Nam. Hanoi: Climate Change Research Group, Hanoi University of Public Health; 2018. |
|  | 26)     Lines 112-3: “support vector regression” is repeated in the list.  27) Line 123: “of one” sounds strange  28)     Line 127: should say “remains” instead of “remain”. | 26) Amended.  27) “of one” changed to “of a DF early-warning system”  28) Amended. |
|  | 30)     There is a recurrent mentioning of “this is the first paper” or “this is the first time”. I value highlighting the novelties of the work, but this does not need to be done twice in the same paragraph (for instance, lines 135-46). Also, novelty should be highlighted in the context of the advantages it brings, not solely on being the first. | Thank you, this was edited accordingly. |
| **Reviewer 5** | No comment |  |
| **Reviewer 6** | 1.      After lines 80 and 81, define reported cases whether cases suspected or confirmed are included. 2.      Lines 110 and 113, it seems that “support vector regression” is repeated. 3.    Lines 138, 139, please add “out of 69” after “20”, this gives a better scope of the study. | 1) Reported cases were confirmed cases. We’ve made this clearer in the revised version.  2) Amended.  3) Amended |
|  | 5.      Line 141, please provide if there is any data on which of the 12 climate factors used as predictors in your modelling, were the most frequently associated with better prediction outcomes. | For all prediction models, using too many data features will often lead to the degradation of overall performance due to inconsistent or overfitting problems. Hence, for each province, we use the Recursive Feature Elimination method to choose two most relevant climate factors to the DF rate. These two factors are used as input for prediction models. List of associated climate factors for each province can be found in Table S1. |
|  | 6.      Line 142, consider adding the actual time frame for short and long-term DF incidence, 1 month and 3 months? | Thank you, 1 month and 3 months were added as the actual time frame. |
|  | Lines 127 and 128, the authors state that the northern and central region of Vietnam “have not been touched”, this implies that no DF prediction models were assessed in both areas, however, have you considered the study by Colon-Gonzales F., et al. 2021 PLos Med study? this study has evaluated predicting modes for DF in Vietnam and tested their model in the entire country. | Thank you for bringing this paper to our attention, we had not come across it before. We believe that our work and this work were conducted at the same time.  The Colon-Gonzales F., et al. (2021) paper has now been briefly mentioned in the introduction, with a more detailed discussion in the discussion section. |
| **Reviewer 7** | No comment |  |
| **3.** | **Materials and methods** |  |
| **Reviewer 1** | I’ll appreciate if you can explain why climate factors including temperature, precipitation, humidity, evaporation, and sunshine hours were chosen for analysis, since there were a range of climate factors, as the article cited in the introduction part. Many factors, such as wind speed and air pressure, were excluded | The climate factors including temperature, precipitation, humidity, evaporation, and sunshine hours were chosen for analysis since these factors have been shown/thought to link with *Aedes* mosquito breeding/behaviors/survival while wind speed has been shown to inversely correlate with DF incidence, and air pressure has been used in previous prediction models. |
| **Reviewer 2** | The study design is appropriate and clearly stated the objective.  Comprehensive retrospective data is used and split into training and testing subsets, which was appropriate for testing the hypothesis. | Thank you. |
| **Reviewer 3** | The study population is made up of data from 1997 to 2016, which is adequate. In general, the proposed statistical analysis is consistent with the objective of the study. | Thank you. |
|  | In general, the article is consistent and accurate. I recommend changing the brackets for the parentheses in line 206, [0,1] to (0,1). I recommend a "Minor Review" | Amended |
|  | The objective of the study is clear when proposing a prediction model based on climatic variables. I consider it necessary to specify the study design. It is easy to understand that it is a prospective longitudinal ecological study. However, the research group needs to define its study. | Thank you, the study design “retrospective ecological study” was added as advised. |
| **Reviewer 4** | 3) The data employed is very adequate. There is extensive case data all over Vietnam for a considerable span of time (20 years).  4) The tools employed are adequate and respond to the state-of-the-art in machine-learning. | Thank you. |
|  | 5) Analyses could be improved if varying training sets were used to test how sensitive the performance and results of the models are to the training and test data. | It is an interesting point. For prediction methods, people typically want to have as much data for training as possible (especially for data hungry models like Deep Learning ones), hoping that this can lead to better prediction accuracy. However, it is not entirely correct. More data can lead to overfit and a few bad ones can ruin the whole model, leading to worse results. Currently, there is no known relationship between the size of training and test data (either in theory or in practice). Actually, in our preliminary studies, we have conducted experiments using different test data (from 1 to 5 years). The same conclusion is drawn. |
|  | 6) k=3 looks rather short. There is no justification for this choice. It would strengthen the results showing the performance for a range of k, say 2 to 6, for different lookback window lengths. | Our algorithm is designed to cope with any values of k. Typically, we can try to predict 6 months or a year ahead. However, besides climate factors, DF rate is affected by many other non-climate ones such as human behaviour, travel patterns, or health policy. These complicated factors are hard to be collected and input into prediction models. In our current era, due to the developments of model communication and transportation technologies as well as the severe global climate changes, the factors also change rapidly, making further ahead predictions like 6 months less meaningful. Hence, in our paper, we choose to show the results of k = 3 since 3 months is a good enough time frame for public health preparations and responses for any DF risks.  S4 Figure shows the results of 6-steps ahead prediction for Hanoi. As we can see, the further we want to predict, the lower the prediction accuracy in general. It shows the same conclusion with the 3 month ahead prediction reported in our paper. The same conclusion is observed for all other provinces. |
|  | 7) What criteria were used to choose the structure and parameters of the models? For instance, lines 246-7, or 287-9. Please, explain in text. | Structures and parameters were chosen by hand. For each province, we try to turn the model independently. |
|  | 8) An explanation about the different information captured by MAE and RMSE would help interpreting the results. Something on this regard appears in the midst of the discussion (lines 455-6), but a richer explanation should appear in Methods. Additionally, it would be informative to provide criteria on how to understand values of these statistics, besides comparing the relative values. | Thank you, we have added more detailed descriptions of MAE and RMSE in the methods section. |
|  | 13) Handling of missing data: why were missing data not interpolated between adjacent months with data? This seems more appropriate than extrapolating from another year, particularly for dengue, that has such interannual variability. Please, better justify in the text or change the method. Just mentioning “We found out that this scheme brings better prediction performance” seems insufficient. | There are many different ways to deal with missing data. Trying all of them for all methods and all provinces is infeasible. Thus, in our work, we use a few provinces like Hanoi to study the impact of imputation methods. We try different methods such as 0 imputation, mean imputation, etc… Moreover, missing data in our work only occurs rarely in climate data (not DF data). Thus, we try to impute them by using minimum values of the same months of the last 2 years (to avoid effects of climate changes). Preliminary results show that this scheme works well for studied provinces. Thus, we apply it for all other provinces. However, as the review pointed out, there may exist better imputation methods. A comprehensive comparison among different methods, prediction models and provinces can be an interesting future work. |
|  | - What “one step” or “multi step” means should be explained in Methods (it is explained in the middle of the results (line 381), after being used paragraphs before (line 294)). | Thank you, we have added the following text in the “Performance evaluation” section of the methods: “Models were evaluated for predictions made one to three months (steps) in advance. Multi-step prediction refers to forecasts made more than one month in advance.” |
|  | 34)     Line 186: “sunshine” refers to daylight or having no clouds? Please, explain in the text. | Sunshine hours is the number of hours with the intensity of direct solar radiation reaching the surface (2m above the ground) with a value equal to or greater than 0.2 calories/cm^2^ minute). Thus, if there are thin clouds, ensuring that solar radiation through is greater than 0.2 calories/cm^2^ minute, it will still be counted as sunny time. Sunshine time is measured with a Campbell–Stokes recorder and is placed in a meteorological garden, where other factors such as temperature, precipitation are measured. |
|  | 33)     Be consistent in how regions are named. E.g., “Middle” or “Central”?  35)     Line 185 and elsewhere: I prefer referring to “weather” instead of “climate” in this context, as climate refers to the average weather patterns along decades, while here you are addressing the particular meteorological conditions of each month. 36)     Lines 213-4: how was overfitting assessed?  31)     Lines 157, 159, and elsewhere: the standard is to leave a space between a magnitude and its unit. 32)     Caption of Fig 1: “Vietnam” is unnecessarily repeated. | 33) “Central” is now used consistently.  35)     Amended.  36)     Amended.  31) Spaces have been added between magnitudes and units. As °C was not mentioned between lines 157 and 159, the comment was assumed not to refer to degrees.  32) Amended. |
| **Reviewer 5** | In this manuscript, the authors explored the potential of using deep learning methods to predict dengue monthly incidence. They compared the performance of multiple deep learning models, including convolutional neural network (CNN), Transformer, long short-term memory (LSTM), and attention mechanism-enhanced LSTM (LSTM-ATT) models. They used the Root Mean Square Error (RMSE) and Mean Absolute Error (MAE) indexes to compare these models. I have below comments and suggestions:  The authors may wish to compare using deep learning models versus using simpler regression models. | Traditional statistical and machine learning models (Poisson regression, XGBoost, support vector regression, linear support vector regression, and SARIMA) have been added and discussed for comparison. |
|  |  |  |
|  | The authors have clearly defined the objectives of the study and have proposed clear testable hypothesis. The study design appropriately addressed their objectives and used an appropriate population to test the hypothesis. However, additional information on the study areas, specific metrics for predictor factors ranking, and information on metrics for prediction methods should be provided. | Thank you, we have revised this. |
|  | In page 5, the authors mentioned many other methods that are different from deep learning models. If I understand correctly, the basic idea of deep learning models is to perform complex regression to capture the complex dependency between data and predictors. As the authors have mentioned other types of simpler regression models, such as Poisson regression models, generalized additive models, I’d like to suggest the authors to compare the performance of using complex deep learning models versus that of using simpler regression models. This comparison will let us know whether using complex deep learning models can perform better than using simpler regression models. | Traditional statistical and machine learning models (Poisson regression, XGBoost, support vector regression, linear support vector regression, and SARIMA) have been added and discussed for comparison. Results show that the traditional methods acquire worse performance than DL methods on this study. |
|  | For predictors, the authors mainly considered climate factors. However, population immunity is an important predictor for dengue severe disease. For example, primary infection is known as the best predictor of developing severe dengue. The authors may wish to include population immunity as an important predictor. | Yes, as you have pointed out that population immunity is an important predictor for dengue severe disease. Primary infection is known as the best predictor of developing severe dengue. However, these data were not available in Vietnam in the past decades, so we could not include population immunity as an important predictor in this study. |
|  | Is it possible to apply your method to dengue data in year 2020 and 2021? | The data are not yet available. |
| **Reviewer 6** | This study indicates that prediction robustness was increase by analysing 12 meteorological factors. Although, those factors were ranked per province, and the top 2 factors were selected/province to be imputed into testing models. It is not clear how the ranks were defined, were R2 coefficient of determination estimated? | This was briefly mentioned in the results under “Data pre-processing”. To improve clarity, a more detailed description has been added to the methods under “Feature selection”. |
|  | A brief description of the “outbreak prediction accuracy” method and the statistical relevance is missing, although the first result presented in Fig 1 depicts predicted incidences vs. real incidence as part of the 1-step forecasting accuracy, there is need of statistical values, e.g. 95% credible intervals, or IC values for the real incidence line depicted on the graphs. | We set an epidemic threshold for each province by using the monthly mean and standard deviation of incidence rates for that province. An outbreak month is defined by an incidence rate exceeding the mean by n standard deviation(s). We set n = 1 in our study to capture both medium and large outbreak months. These information have been clarified in the paper.  Moreover, we only check if the algorithm can detect an outbreak correctly using the predicted result. Thus, we do not have confident interval values. |
|  | The precision, accuracy, sensitivity, and specificity of multi-step ahead epidemic prediction methods should be described here in the methods, this information is given at the end of results when describing fig 11. It will help the reader to get this information earlier that at the result section. | Precision, accuracy, sensitivity, and specificity have been defined in the methods, now. |
|  | Line 164, a definition of “incident cases” is needed to clarify if the case counts included suspected cases or confirmed DF cases. evaluated. | Thank you, in this sentence, we meant confirmed DF cases, and this was revised accordingly to make it clearer. |
|  | Lines 185-187, authors indicate that 12 meteorological factors were included in the study, but a clear description of the five types of measures would improve clarity, e.g. temperatures were measured as minimal, or maximal temperatures, where those “air temperature measured 2 meter above the grown, etc.? | Thank you, details about the 12 meteorological factors and their units are provided in the revised Table, which included an additional column showing the clear description of the factors |
|  | Line 202, the “datasets had missing few data points”, in which provinces? Please indicate which provinces, have these missing points impacted negatively on the data even despite of the normalization the authors performed? | Some provinces like Quang Nam has a few missing data points in some climate factors. However, the missing ratio is very low. Thus, the effect on algorithm performance is negligible, especially when chosen climate factors may not contain any missing values. |
|  | Line 206, please expand on the “different features and different value ranges” to improve clarity. | We have defined what we mean by different features and value ranges, with examples. |
|  | Line 212, 213, you referenced how the 12 meteorological factors were ranked per province, to define 2 top ranked factors. Accordingly, the supplemental S1 Table, the top 2 factors selected/province are listed, but a metric of the rank values are not provided. This information will add clarity to the results obtained. | For each province, all climate factors are assessed using the Recursive Feature Elimination (RFE) method and the top 2 factors are selected as inputs for prediction models. The full list of ranked climate factors for each province is shown in Table S1. |
|  | Line 235, please add “in Malaysia” after “Kuala Lumpur” for clarity. | Amended |
| **Reviewer 7** | The study shows for the first time the use of deep learning models for climate-based Dengue Fever forecasting. The methods are well described with details. The statistical analysis was well performed in my opinion. | Thank you |
| **4.** | **Results** |  |
| **Reviewer 1** | The yearly data shown in Fig 1 barely have relations with the result, so it seems unnecessary. | Figure 1 shows the overall DF situation in different provinces and regions of Vietnam. As we see, Middle and South regions have more severe DF rates. We expect that it will enrich the understanding of the overall DF pictures in Vietnam. |
|  | Fig 2 can be elucidated in words. | Description of Fig 2 has been revised in Section 2.3 in more details. |
|  | Only the results of 6 provinces were manifested in Fig 6. Please explain what dose “the other provinces do not convey additional information”, in line 298-300, mean? It would be advantageous if a supplementary figure could be presented. | A supplementary figure of all provinces has been added to give a clear overview of the entire results. |
|  | The actual outbreak of 15 provinces is zero in Fig 9. So most of precision and accuracy are zero. It makes the conclusion about the prediction ability of LSTM-ATT not so persuading. | For the 15 provinces referred to, the precision and sensitivity are undefined (division by zero), not zero. The accuracy, precision, sensitivity, and specificity formulas added to the methods section should clear this up. Accuracy refers to months correctly assigned as outbreak or non-outbreak, so it is not 0 for any provinces. Precision and sensitivity concern outbreaks and predicted outbreaks only, but these are not zero in any provinces except Thai Binh (sensitivity of 0). |
|  | True positives depend on rate in line 354, while false positives depend on number in line 356. It might be inconsistent. | Number changed to rate. |
|  | Fig 10 shows the multi-step ahead predictions results of three provinces. It will be appreciated if the results of the other 17 provinces could be put in the supplementary figure. | A supplementary figure of the multi-step results for all 20 provinces has been added. |
| **Reviewer 2** | The analysis presented match the analysis plan  The results are clearly and completely presented The figures are sufficient quality for clarity | Thank you. |
|  | Some figures can be transferred to supplementary files. Accept with Minor Revisions. | Thank you. |
| **Reviewer 3** | - The results obtained in the study coincide with the proposed analysis plan. On the other hand, figure 2 is not very informative, the process could be written in the document. Figure 2 is reversed. The other images are adequate. | Thank you. We will add the process for Fig 2 and reverse it. |
| **Reviewer 4** | 2) I would find very informative to see a figure (maybe as supplementary, or replacing Fig 1) displaying the complete epidemic curves for each province along the 20 years. | A supplementary figure of the complete epidemic curves for all provinces has been added and mentioned. |
|  | 9)  I am not sure it is worth devoting space to Figs 2-5 in the main text. | Fig 2 is to explain the whole data processing and prediction in our work. However, we are happy to remove it if the reviewers still request. Figs 3 to 5 were added to make the paper self-explained (for reader who are not familiar with DL methods), but have been removed. |
|  | 10) Results contain some description of the methods that should be moved to the method section (e.g., lines 346-56; 369-77). | The descriptions of the methods have been moved to the methods section. |
|  | 11)  Please, for clarity avoid using the same name for different variables. E.g., k used for the prediction window and for SDs for outbreak definition. | Changed so that *k* is used for prediction window and *n* is used for SDs. |
|  | 12) I would not call an “outbreak” each month above the threshold. I find it counterintuitive because usually an outbreak is considered as the whole “peak” that may exceed a month. Instead, referring to “epidemic months” or “outbreak months” could be more eloquent. | This has been changed for most references to outbreak month predictions, except when referring to the general concepts of outbreaks and outbreak forecasting. |
|  | 16) Results for all provinces could be provided in the supplementary material. | Results for all provinces are now provided in the supplementary materials. |
|  | 17) What “one step” or “multi step” means should be explained in Methods (it is explained in the middle of the results (line 381), after being used paragraphs before (line 294)). | We have added the following text in the “Performance evaluation” section of the methods: “Models were evaluated for predictions made one to three months (steps) in advance. Multi-step prediction refers to forecasts made more than one month in advance.” |
|  | 18) Most results (and figures) refer to predictions one month ahead. Initially, this is not clear at all since authors refer to “one-step” without timely explaining what a “step” is. The explanation appears paragraphs after in the midst of the results (line 381). However, in Methods (lines 240-1), it is stated that k=3 “in this paper” which, as explained there, means that predictions are made 3 months in advance. Predicting one month in advance does not look of great utility for implementing control measures. Also, generally transmission varies rather smoothly between adjacent months (Fig 6 for instance). Therefore, predicting one month in advance should not be a very difficult task, even without such complex methods. Instead, predicting 3 or, even better, 4-6 months in advance is not straight-forward and can indeed be of help for vector-control programs. It would be very informative seeing something like Fig 10(bottom) but at the very beginning of Results, for all provinces (at least in supplementary results), and including larger k’s. | Supplementary figures have been added to show predictions in all provinces for all models, to show 1–3 month ahead predictions in all provinces by LSTM-ATT, and to show up to 6 month ahead predictions in Hanoi by LSTM-ATT. Further discussion on long-term prediction can be found in the above response too. |
|  | 19) As currently presented, figures have a poor resolution, even when downloaded. | We try to improve the Figure as much as possible. |
| **Reviewer 5** | (No comment) |  |
| **Reviewer 6** | 1. Most of the results are clearly and completely presented as outlined in the study design, however additional information on some of the results may improve clarity and relevance to the current findings. | Thank you. |
|  | 2. Fig 1 depicts predicted incidences vs. real incidence as part of the 1-step forecasting accuracy, there is need of statistical values, e.g. 95% credible intervals, or IC values for the real incidence line depicted on the graphs. | Unlike some traditional statistical methods such as SARIMA, Deep Learning methods like LSTM cannot provide statistical intervals. |
|  | 3. The Table S1 provide a list of the top 2 geometrical factors that ranked best among 12 factors evaluated per each province. Actual rank values could be added for each selected factor for data completeness. | Table S1 has been amended to show the full rankings of all factors. |
|  | 14.     Lines 298-300, only 6 provinces were selected to illustrate the prediction accuracy data, please explain why those provinces were selected and why the other 14 provinces were not. Additional information is needed to clarify whether the other 14 provinces have similar outcomes or perhaps opposite outcomes, this information will improve clarity.  15.     Lines 300, 301, prediction lines for LSTM an LSTM-ATT are considered to have “very good prediction accuracies”, again, statistical data is needed to understand why it is considered a “very good prediction accuracy”. Although, authors indicate that the MAE and RMSE values (Figure 7) provide the metrics of fig 6, the fitted predicted incidence lines should be compared with the real incidence lines to see whether the predicted lines fall closer or under the 95% credible interval of the real incidence line. | For the main paper, showing all 20 provinces is lengthy. Thus, we randomly choose 6 provinces for showing the results. The full results for all provinces can be found in Supplementary S4 Figure.  15. As we mentioned above, DL methods do not provide confident line like some traditional prediction method such as SARIMA. |
| **Reviewer 7** | The figures and tables presented shows the results with quality and clarity. The authors used different type of graphics according to data. The results are clearly demonstrated. |  |
| **5.** | **Discussion** |  |
| **Reviewer 1** | No comment |  |
| **Reviewer 2** | No comment |  |
| **Reviewer 3** | No comment |  |
| **Reviewer 4** | 38)     Lines 431-9: this paragraph sounds like part of Introduction or a justification in Methods, rather than Discussion. | The paragraph has been moved to after line 117 in the introduction. |
|  | 39)     Line 436: requiring “specific domain knowledge” is not a disadvantage of traditional models and, in fact, any analysis requires specific knowledge (for instance, machine learning methods). | The words, “and requires specific domain knowledge”, have been removed. |
|  | 40)     Lines 452-5 and 472-474: this should be moved to Results; it is not properly discussion. | The referenced lines have been deleted and amended, respectively. |
|  | 41)     Lines 461-9: this paragraph seems out of the scope of this paper as it addresses data/results not shown and it delves into matters not within the objectives and analyses proposed. As is, this paragraph should be removed. | Thank you, we have removed this paragraph. |
|  | 42) “Long term”: what is considered long term? What criteria are used for defining long term? | Typically, most prediction methods only try to predict the next data point. In our case, we design our methods so that they can predict multiple data point at the same time. Since each data point represents a month, our method can predict future values of many months ahead. This is called long-term prediction. |
|  | 43)     Line 480: syntax seems confusing or incorrect. | Syntax has been improved. |
| **Reviewer 5** | No comment |  |
| **Reviewer 6** | 16.     Lines 432- 436, there are 4 limitations of traditional machine learning (ensemble and statistical) models that the authors discuss but no reference is provided. Please add references for these 4 limitations. | We have removed this part. |
|  | 17.     Lines 489-490, perhaps the study conducted by Colon-Gonzales F., et al. (2021 PLos Med), should be considered as they tested short (1 month) and long term (3 and 6 months) prediction models in the 63 provinces of Vietnam from over 19 years, from 2002-2020. | A discussion of the Colon-Gonzales F., et al. (2021) paper has been added. |
|  | 18.     Lines 516-518, since missing cases and climate data are considered potential confounding factors, how do the authors address these factors in this study? Similarly, differences of performance have been found in some provinces compared with the rest, but a discussion on how the authors overcome this limitation is not provided. | We clarify how to deal with missing climate data in Section 2.3. |
| **Reviewer 7** | No comment |  |
| **6.** | **Conclusion** |  |
| **Reviewer 1** | No comment |  |
| **Reviewer 2** | No comment |  |
| **Reviewer 3** | No comment |  |
| **Reviewer 4** | 44)     Conclusion paragraph: it looks like a summary, rather than a conclusion (except for lines 534-8). A further emphasis on the implications of the paper, beyond what has already been said in the rest of the discussion, could turn this into a proper conclusion and enrich the work. | We rewrite the conclusion section following the suggestions of reviewers. |
| **Reviewer 5** | No comment |  |
| **Reviewer 6** | 19.     Lines 530-531, the authors indicate that the three regions studied in Vietnam have significant differences in geographical and climate conditions, however, there is lack of information on these “geographical and climate conditions” for these three regions through the manuscript. Authors should consider providing this information early when describing the study in material and methods section. | Thank you, we added this in the 2.1 section. |
| **Reviewer 7** | No comment |  |
| **7.** | **Other comments** |  |
| **Reviewer 1** | No further comments |  |
| **Reviewer 2** | No further comments |  |
| **Reviewer 3** | No further comments |  |
| **Reviewer 4** | 14) There are no ethical concerns.  15) Data are not made available. Given that the data used does not imply any privacy concern (because it is aggregated at a province level), I would expect that authors could meet the journal spirit of increasing data availability. Additionally, no specific web address or e-mail is given to ask for the data, as indicated in the journal guidelines.  45)     Line 550: “a the” seems an error. 46)     Reference formatting is not homogenous, nor it complies always with journal guidelines. | This study was approved and managed by the Hanoi University of Public Health. The study only involved analysing secondary data on dengue fever cases and climate factors. No human participants were actually involved in this study. Thus, ethical approval was not required.  We need to pay for climate data, so are not made available for everyone. If any scientists wish to have access to the data, please email the corresponding author and she need to ask for permission from the 2 institutions before sending data to the interested scientists.  45) Amended  46) I am not exactly sure where the issues with the reference formatting are. I used Zotero’s PLoS NTD formatter, and I could not see any major issues looking through. I added a missing publisher for one report, and changed “Available” to “Available from” for URLs. |
| **Reviewer 5** | No further comments |  |
| **Reviewer 6** | References  20.     Under reference #3. Change “Organisation” for “Organization” | 20) Amended |
| **Reviewer 7** | I have no further recommendations to the authors or Editor. The paper is well written. | Thank you! |

Thank you all so much for your valuable comments, which helped us to greatly improve the quality of the manuscript.
