# Supplementary material for: Deep learning models for forecasting dengue fever based on climate data in Vietnam
Source: PLoS Negl Trop Dis. 2022 Jun 13;16(6):e0010509. doi: 10.1371/journal.pntd.0010509 (PMC9232166; doi:10.1371/journal.pntd.0010509)
Supplement: Attachment — Submitted filename: Responses to reviewers comments 4 March 2022.docx [file pntd.0010509.s012.docx]

**RESPONSES TO REVIEWERS’ COMMENTS**

**Deep learning models for forecasting dengue fever based on climate data in Vietnam**

Van-Hau Nguyen^1^, Tran Thi Tuyet Hanh^2^*, James Mulhall^3^, Hoang Van Minh^2^, Trung Q. Duong^3*^, Van-Chien Nguyen^4^, Nguyen Thi Trang Nhung^2^, Vu Hoang Lan^2^, Hoang Ba Minh^5^, Do Cuong^6^, Nguyen Ngoc Bich^2^, Nguyen Huu Quyen^7^, Tran Nu Quy Linh^8^, Nguyen Thi Tho^9^, Ngu Duy Nghia^9^, Le Van Quoc Anh^10^, Diep Phan^11^, Quoc Viet Hung Nguyen^8^, Son T. Mai^4^

| **No.** | **Reviewers’ comments** | **Responses from authors** |
| --- | --- | --- |
| **1.** |  |  |
| **General comment** | - The language needs to be checked carefully. - Please upload the analyzed data as a supplementary file for easy reproducibility of the reported findings. This will be a criteria for acceptance. - Please write the section name and lines/pages number for every change you make. | -Thank you, the author team, especially James Mulhall from Queen’s University Belfast, has carefully revised the manuscript.  - We have uploaded the analyzed data as a supplementary file for easy reproducibility of the reported findings.  - The section name and lines/pages number for every change made included:  **Author Summary**  Line 58: cases changed to rates  Line 59: response changed to responses  Line 67: "it" added  **Introduction**  Line 73: "populations are specially adapted to urban environments due to" changed to "are particularly suited to urban environments, where there is"  Line 107: "used" added  Line 108: comma changed to semi-colon  Line 139: "Besides these studies" removed  Line 147: hyphen added to mechanism-enhanced  Line 148: case numbers changed to rates  **Methods**  Line 274: "outbreak months to the total number of non-outbreak" changed to "normal months to the total number of normal"  Line 354: dengue fever changed to DF  **Results**  Line 381: XGBoost changed to XGB  Line 391: "for" removed  **Discussion**  Line 493: hyphen added to large-scale  Line 592: "or provincial" added |
| **Methods** | Reviewer #2: Yes Reviewer #4: (No Response) Reviewer #5: (No Response) Reviewer #6: Yes, objectives and study design are clear and appropriate. There are no concerns about ethical or regulatory requirements. Reviewer #7: The methods are clearly described and testing in municipal scale instead of the entire country was very important to find a model of deep learning methodologies to associate environmental parameters with dengue fever occurrence. | Thank you! |
| **Results** | Reviewer #2: Authors fairly incorporated all the comments from reviewers Reviewer #4: (No Response) Reviewer #5: (No Response) Reviewer #6: Results are now more clear and complete. Authors have included suggested modifications. Reviewer #7: The results are very well presented with sufficient tables and images. | Thank you! |
| **Conclusions** | Reviewer #2: Authors fairly incorporated all the comments from reviewers Reviewer #4: (No Response) Reviewer #5: (No Response) Reviewer #6: Conclusions are supported by the data, suggested modifications to methods, results, and discussion session now support the conclusions. Reviewer #7: Conclusions were addressed to public health relevance and is supported by the presented data. | Thank you! |
| Editorial and Data Presentation Modifications? | Reviewer #2: Accept Reviewer #4: (No Response) Reviewer #5: (No Response) Reviewer #6: None Reviewer #7: No recommendations | Thank you! |
| Summary and General Comments | Reviewer #2: (No Response) Reviewer #4: The points in my review were adequately addressed. I thank the authors for the effort and clarity in responding the numerous points. Reviewer #5: (No Response) Reviewer #6: The authors have appropriately addressed most of the observations and have incorporated modifications suggested throughout the manuscript. This manuscript if accepted for publication can be a relevant contribution to the community, the forecasting models tested in this work have the potential to enhance public health responses to in the control of climate-impacted diseases. Reviewer #7: The presented work is original and enable prediction of dengue fever outbreaks with a time of three months. The regional scale used is original and enabled the important findings of the work. | Thank you! |

Thank you all so much for your valuable comments from the 1^st^ revision round and for accepting our manuscript. Your comments helped us to greatly improve the quality of the manuscript.
